# Vehicle State Estimation Combining Physics-Informed Neural Network and Unscented Kalman Filtering on Manifolds

**DOI:** 10.3390/s23156665

**Published:** 2023-07-25

**Authors:** Chenkai Tan, Yingfeng Cai, Hai Wang, Xiaoqiang Sun, Long Chen

**Affiliations:** 1 Automotive Engineering Research Institute, Jiangsu University, Zhenjiang 212013, China; 2112104008@stmail.ujs.edu.cn (C.T.);; 2 School of Automotive and Traffic Engineering, Jiangsu University, Zhenjiang 212013, China

**Keywords:** IMU calibration, unscented Kalman filtering on manifolds, physics-informed neural network, vehicle state estimation, multi-sensor fusion

## Abstract

This paper proposes a novel vehicle state estimation (VSE) method that combines a physics-informed neural network (PINN) and an unscented Kalman filter on manifolds (UKF-M). This VSE aimed to achieve inertial measurement unit (IMU) calibration and provide comprehensive information on the vehicle’s dynamic state. The proposed method leverages a PINN to eliminate IMU drift by constraining the loss function with ordinary differential equations (ODEs). Then, the UKF-M is used to estimate the 3D attitude, velocity, and position of the vehicle more accurately using a six-degrees-of-freedom vehicle model. Experimental results demonstrate that the proposed PINN method can learn from multiple sensors and reduce the impact of sensor biases by constraining the ODEs without affecting the sensor characteristics. Compared to the UKF-M algorithm alone, our VSE can better estimate vehicle states. The proposed method has the potential to automatically reduce the impact of sensor drift during vehicle operation, making it more suitable for real-world applications.

## 1. Introduction

The rapid development of sensor technology and intelligent transportation systems [1] in recent years has led to the introduction of new vehicle chassis subsystems by original equipment manufacturers [2]. These subsystems improve specific vehicle performance, making driving more efficient and effective. Vehicle chassis subsystems not only make driving more convenient [3], but they also make the vehicle a complex autonomous system. The optimal coordination of chassis system (OCCS) [4] is applied to coordinate different complementary chassis subsystems. However, accurate vehicle state information is required for chassis coordinated control in order to correctly coordinate subsystems and identify driving conditions [5]. Furthermore, sensor noise can affect the accuracy, reliability, and continuity of vehicle state information. Extensive research [6,7] has been conducted to investigate various estimation algorithms based on cost-effective sensors and available measurements.

With the development of sensor technology, the use of vehicle control sensors such as the global navigation satellite system (GNSS) and the inertial measurement unit (IMU) has increased. In the OCCS, the IMU plays a crucial role in measuring the angular rate and acceleration of the vehicle body, enabling accurate calculations of the vehicle's attitude, velocity, and position [8]. However, due to installation errors [9] or coupling effects between the IMU and vehicle motion [10], IMU misalignment is inevitable. Indirect state estimation methods [9,10] are proposed to mitigate the drift of IMUs. To achieve advanced control, such as the OCCS, the coordinator requires multiple advanced sensor inputs and more estimation outputs, primarily to avoid conflicts downstream in subsystems [2]. This has motivated us to develop a method that leverages the relationships between advanced sensors to eliminate IMU drift.

The extended Kalman filter (EKF [11]) and unscented Kalman filter (UKF [12]) are commonly used for optimal integration between the GNSS and inertial navigation system (INS). The unscented Kalman filter on manifolds (UKF-M) [13] is a novel filtering algorithm that provides more accurate and robust navigation estimates. Compared with the existing state-of-the-art integrated navigation algorithms, the UKF-M-based integrated navigation estimation algorithm [14] has higher accuracy and faster convergence speed. These methods incorporate various vehicle/tire models and real-time states [15] to handle disturbances and noise. However, the local linearization operation of EKF/UKF can introduce significant estimation errors [7]. Additionally, sensor offset can cause integration errors in the INS/GNSS model [16].

A virtual sensor (VS) [17] can be used to replace a redundant sensor, which can mitigate sensor noise. A VS is a type of software sensor that can integrate multiple data sources to improve system reliability. Kim et al. [18] combined the adaptive Kalman filter with a deep neural network (DNN) to estimate the sideslip angle. The proposed model utilized the DNN output as a VS. Combining the EKF/UKF model with the DNN-based VS, this model not only provided accurate estimates of the sideslip angle but also quantified the uncertainty associated with the estimation. In another study, Kim et al. [19] used a long short-term memory (LSTM) network to filter the noise and bias of the original sensor data. Leandro et al. [20] combined a neural network and a Kalman filter to estimate the vehicle's roll angle. The neural network output was used as the pseudo-roll angle to build the Kalman module. Soriano et al. [21] proposed a neural network-based calibration method for a two-axis accelerometer. Their experimental results demonstrated that the accelerometer error model based on the neural network had better accuracy and robustness than the explicit accelerometer error model method. 

The data-driven vehicle model [18,19,20] serves as an approach for establishing VS. These models utilize a data-driven approach to estimate vehicle states by leveraging the hidden relationships between them. In the development of data-driven vehicle dynamics models, the linear time-invariant (LTI) state-space model [22,23,24,25] is commonly employed. Experimental results [23,24,25] demonstrated that the LTI-based data-driven vehicle model outperformed comparative vehicle dynamics models. However, these data-driven vehicle models [18,19,20,23,24,25] rely on the assumption that the selected supervised learning vehicle states accurately represent the true vehicle states. This assumption can impact the effectiveness of online learning in these models. Additionally, due to the influence of sensor noise [26], neural network-based models may introduce errors without physics-based models.

The physics-informed neural network (PINN) [27] is a novel deep learning method that integrates domain-specific knowledge into a neural network architecture. Xu et al. [28] proposed a PINN-based model for unmanned surface vehicle dynamics. Compared with traditional neural networks, their PINN-based unmanned surface vehicle dynamics model had better prediction accuracy for the sway and surge velocity and rotation speed. Franklin et al. [29] used PINN as a hybrid virtual sensor to estimate the flow metering in oil wells. Wong et al. [30] demonstrated that PINN can effectively mitigate the impact of noise in data originating from low-quality sensors. In state estimation, combining PINN with a state-space model formulation can avoid computationally costly time integration [31]. 

In this study, we propose a vehicle state estimation (VSE) method that combines PINN and UKF-M (PINN UKFM). The contributions of the paper can be divided into two main parts.

- We use PINN as a data-driven vehicle dynamics model to establish a VS, using the IMU calibration values as the output. By leveraging the loss calculation method of PINN, the vehicle dynamics can be integrated with the data-driven model, enabling the incorporation of information from multiple sensor sources during vehicle operation. The experimental results in a real vehicle platform indicated that the PINN-based model effectively integrates multiple sensor inputs to achieve improved estimation of the vehicle’s state, surpassing both the physical and neural network-based models.
- Based on the IMU calibration values, we utilize the UKF-M algorithm to estimate the altitude, velocity, and position of the vehicle. By fusing data from multiple sensors, PINN UKFM provided accurate and comprehensive vehicle states that included six-dimensional vehicle dynamics, 3D attitude, speed, and position, which can be used in various vehicle dynamic control systems. For example, the six-dimensional vehicle dynamics can define the chassis motion, which can be used in OCCS. The 3D position can be applied to vehicle navigation in a GNSS-denied environment. The experimental results indicated that the PINN-based model can effectively incorporate multiple sensor inputs to mitigate IMU biases and enhance the accuracy of the existing state-of-the-art integrated navigation algorithm, UKF-M.

The rest of work is organized as follows: Section 2 introduces the vehicle model, the PINN UKFM sensor states, and defines the PINN UKFM problem. Next, Section 3 introduces the proposed PINN UKFM algorithm. In Section 4, the proposed method is tested using realistic vehicle data. Rainy weather is used as an experimental condition as it reduces the road adhesion coefficient and increases the nonlinearity of vehicle dynamics. Finally, Section 5 presents the conclusions of this paper.

## 2. Estimation Problem

### 2.1. The Vehicle Model

**Figure 1:**
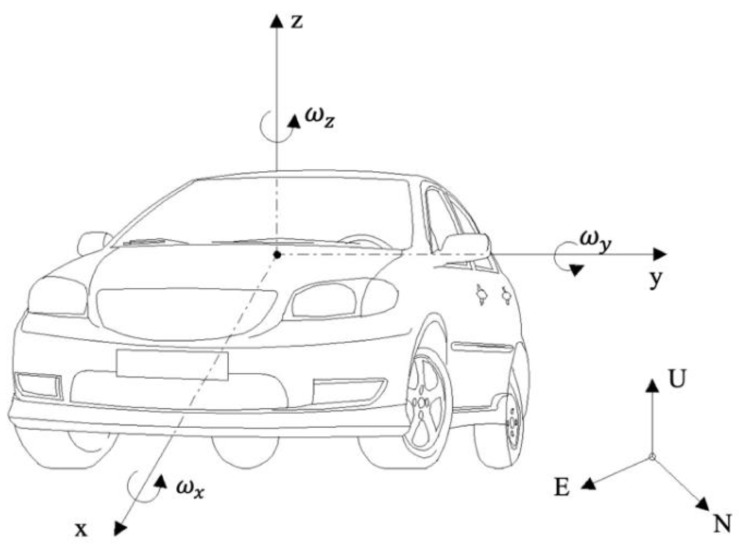
The 6-DoF vehicle model coordinates.

The PINN UKFM algorithm used a six-degrees-of-freedom (6-DoF) kinematic vehicle model [32] to obtain accurate state estimates. As shown in Figure 1, the model could define the chassis movement [33], which included the navigation and vehicle body coordinates [5]. The starting point of the navigation coordinates was defined as the track start. The navigation coordinates consisted of three variables: E (east), N (north), and U (upward).

The vehicle body’s coordinate origin point was located at its center of mass and the right-hand rule was used. The x-, y-, and z-directions pointed forward, left, and upward, respectively. Acceleration and velocity could be broken down into longitudinal acceleration $a_{x}$/velocity $v_{x}$, lateral acceleration $a_{y}$/velocity $v_{y}$, and vertical acceleration $a_{z}$/velocity $v_{z}$. The vehicle’s direction angle was defined as the rolling angle (around x, roll rate $\omega_{x}$), pitching angle (around y, pitch rate $\omega_{y}$), and heading angle (around z, yaw rate $\omega_{z}$). Finally, other vehicle states could be extrapolated from these. For example, the sideslip angle β could be calculated through the following trigonometric operation:

(1) $$\beta =\tan^{-1}(\frac{v_{y}}{v_{x}})$$

### 2.2. The Sensor States

In the experiment, the vehicle had multiple sensors, including the GNSS, IMU, measurement steering wheels (MSW), wheel force transducers (WFT), 2-axis optical sensors (S-Motion), and a monocular camera. The experimental platform structure is presented in Section 4.1. Due to the real-time computation requirement, the signals from the monocular camera were not used in the PINN UKFM algorithm. The sensor inputs of PINN UKFM are introduced in Table 1.

The symbols E and N represented easting and northing in the navigation coordinates. Using the universal transverse mercator (UTM) and the navigation starting point, easting and northing were transformed from the GNSS coordinates into the navigation coordinates. The UTM velocity was calculated from navigation coordinates as:

(2) $$v_{gps}=\frac{\sqrt{{\left(E^{t}-E^{t-1}\right)}^{2}+{\left(N^{t}-N^{t-1}\right)}^{2}+{\left(U^{t}-U^{t-1}\right)}^{2}}}{dt}$$

where *dt* is a single GNSS interval, and *t* and *t* − 1 are GNSS coordinate timestamps. 

### 2.3. Problem Definition

The PINN-based VS was defined as a time-series forecasting model in which sensor signals were taken as discrete variables. Assuming that “n” represents the current timestamp, the PINN module input states were defined as:

(3) $$X=\left[X_{n-L},X_{n-L+1},\ldots ,X_{n}\right]\;X_{n}=[X_{n}^{\left(1\right)},X_{n}^{\left(2\right)},\ldots ,X_{n}^{\left(\vartheta \right)}]$$

where n−L is the starting time step, *X* represents the sensor signals shown in Table 1, and ϑ is the number of sensor signals. 

PINN was used as a universal function approximator to achieve IMU calibration. Building upon previous artificial intelligence-based techniques and the integration of the Kalman filter for estimation [23], the calibrated values were called “pseudo-states.” By applying the conservation principles derived from the vehicle dynamics, the pseudo-states satisfied the conservation principles originating from the vehicle dynamics. Therefore, the PINN module output states were defined as:

(4) $${\hat{u}}_{\theta }=[{{\hat{u}}_{\theta }}^{\left(1\right)},{{\hat{u}}_{\theta }}^{\left(2\right)},\ldots ,{{\hat{u}}_{\theta }}^{\left(\iota \right)}]$$

where ${\hat{u}}_{\theta }$ represents the pseudo-states, and ι is the number of pseudo-states. Based on the LTI state space assumption, the pseudo-states were inputted to ODEs to compute the integrated states. The sensor measurements of these integrated states z were represented as:

(5) $$z=[z_{n+1},z_{n+2},\ldots ,z_{n+F}]\;z_{n+1}=[z_{n+1}^{\left(1\right)},z_{n+1}^{\left(2\right)},\ldots ,z_{n+1}^{\left(\kappa \right)}]$$

where $\left[n+1,n+2,\ldots ,n+F\right]$ represent the output timestamps, κ is the number of the integrated states, and n+F is the ending time step.

For better integration with the vehicle control, the authors hypothesized that the vehicle physical model satisfied an LTI state-space model [23,24], which could be defined as:

(6) $$\left\{\begin{array}{c} \dot{x}=Ax_{t}+B_{t}\\ y_{t}=Cx_{t}+D_{t} \end{array}\right.$$

where $x_{t+1}$ is the state variable at next time step, $x_{t}$ is the state variable at current time step, $y_{t}$ represents the output variable at current time step, $B_{t}\mathrm{and}D_{t}$ are disturbances or noise, and $\dot{x}$ denotes the rate of change of the state variable. It should be noted that the system dynamics and output equations did not change over $\left[n+1,n+2,\ldots ,n+F\right]$. 

By utilizing the LTI state-space model, the output of the PINN module could be used to compute the other vehicle states. Based on the ODE constraints, PINN ensured the estimated IMU states satisfied the physical relationships among sensor measurements. 

Next, the pseudo-states were inputted into the UKF-M-based IMU-GNSS sensor-fusion model [34]. Using these pseudo-states, the UKF-M module could estimate both the velocity and position of the vehicle.

## 3. Methodology

### 3.1. Structure of the Proposed VSE

**Figure 2:**
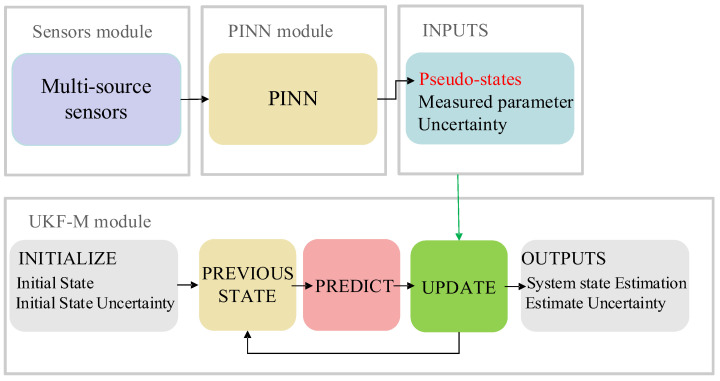
The structure of the proposed VSE.

In recent years, there has been growing interest in the development of multi-sensor systems for vehicle state estimation due to their potential to improve accuracy and robustness in complex environments. The proposed PINN UKFM is one such system, integrating multiple sensors to estimate the vehicle dynamics in real time. The architecture of our proposed PINN UKFM is illustrated in Figure 2 and consisted of three modules: the sensors module, the PINN module, and the UKF-M module.

The sensors module in PINN UKFM consisted of various sensors, such as GNSS, IMU, MSW, WFT, and S-Motion. These sensors provide rich information about the vehicle's motion, including its position, velocity, acceleration, and orientation. For adaptive reduction of data noise, the PINN module was employed to learn the complex, nonlinear relationship between the sensor signals and the filtered vehicle states. Specifically, the PINN module used the time-series sensor signals as input and outputted the corresponding pseudo-states. These pseudo-states were then fed into the UKF-M module, which used a state-space model to estimate the vehicle's position, velocity, and other parameters. By combining the strengths of both PINN and UKF-M, the proposed PINN UKFM achieved accurate and robust vehicle state estimation.

### 3.2. The PINN Module

In practical applications, different sensors may measure data with noise and drift due to their different characteristics and working environments. To obtain accurate vehicle state estimation, we used PINN to integrate the vehicle dynamics into a neural network architecture. The PINN module punished the loss function with ODEs and algebraic equations to make the sensor data consistent with the vehicle dynamics. The PINN module fused the data from multiple sensors to reduce measurement errors and make the data more consistent with the actual vehicle dynamics.

**Figure 3:**
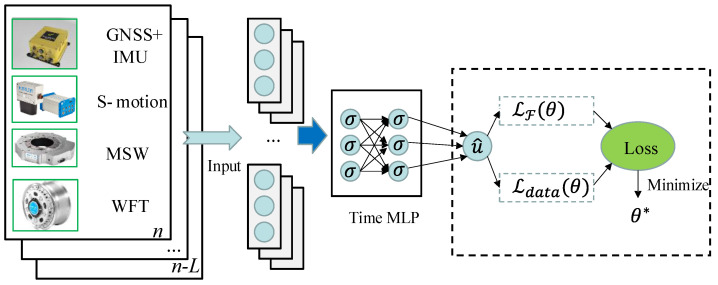
The proposed PINN module.

Therefore, the PINN module found the angle and velocity by integrating the acceleration and angular velocity. Temporal interaction is widely used in establishing data-driven vehicle models [19,25], as it can capture the complex temporal and hidden correlations for better state prediction. Therefore, a temporal model consisting of an encoder layer, a temporal interaction layer, and a decoder layer was proposed, as shown in Figure 3.

The encoder layer was used to embed and encode sensor signal X. Data were mapped into the high dimension through the multilayer perceptron (MLP). The encoder layer was defined as:

(7) $$e_{t}=\sigma (W_{emb}X_{t}+b_{emb})$$

where $e_{t}$ denotes the embedded feature vector. Additionally, the MLP contained the weighting matrix $W_{emb}$, bias term $b_{emb}$, and the rectified linear unit function (ReLU) activation function σ [35].

Since vehicle states have temporal interactions, the temporal interaction layer supposed the temporal interactions of different hidden states. The time dimension of $e_{t}$ was connected:

(8) $$e^{0}=\mathrm{Concat}\left(e_{t}\right),t\in [n-L,n]$$

where the embedded feature vectors were concatenated to form $e^{0}$. Then, the PINN module learned the temporal interaction:

(9) $$e^{l}=\sigma \left(W_{time}^{l}e^{l-1}+b_{time}^{l}\right),\;l=1,\;2,\;\ldots ,s$$

where $e^{l-1}$ and $e^{l}$ are the input and output of layer *l*, respectively.

The decoder layer predicted the pseudo-states as:

(10) $${\hat{u}}_{\theta }=(W_{dec}e^{s}+b_{dec})+u_{past}$$

where $u_{past}$ represents the past measurement of the IMU, and ${\hat{u}}_{\theta }$ refers to the pseudo-states that represent the IMU calibration values. We defined the residual between the pseudo-states and past measurement of the IMU as the drift of the IMU. This structure was similar to the residual block in a residual network [36]. $\left(W_{dec}e^{s}+b_{dec}\right)={\hat{u}}_{\theta }-u_{past}$ represented the latent (hidden) solution of the drift of IMU. The PINN determined the parameter θ of the NN [27] by minimizing the loss function:

(11) $$\theta =\mathrm{argmin}\;L\left(\theta \right)\;L\left(\theta \right)=L_{F}\left(\theta \right)+L_{data}\left(\theta \right)\;L_{data}\left(\theta \right)=\frac{1}{N_{d}}\sum_{i=1}^{N_{d}}{\left|{\hat{u}}_{\theta }\left(X;\theta \right)-u\left(X\right)\right|}^{2}\;L_{F}\left(\theta \right)=\sum_{k=1}^{7}\omega_{k}\cdot [\frac{1}{F}\frac{1}{N_{F}}\sum_{t=n+1}^{n+F}\sum_{i=1}^{N_{F}}{\left|g_{k}\left({\hat{u}}_{\theta }\left(X;\theta \right),X_{n},z_{t}\right)\right|}^{2}]+\sum_{k=8}^{12}\omega_{k}\cdot [\frac{1}{N_{F}}\sum_{i=1}^{N_{F}}{\left|f_{k}\left({\hat{u}}_{\theta }\left(X;\theta \right),X_{n}\right)\right|}^{2}]$$

where $L_{F}\left(\theta \right)$ represents the mean square error of residuals of the physics-based equations; $L_{data}\left(\theta \right)$ represents the mean square error of residuals of the measurement data; $\omega_{1},\omega_{2},\ldots ,\omega_{12}$ denote the weights associated with physical constraints; $N_{d}$ and $N_{F}$ represent the batch size; $u\left(X\right)$ represents the future measurement of the IMU; t represents the output timestamps; F represents the forecast horizon; $g\left({\hat{u}}_{\theta }\left(X;\theta \right),X_{n},t\right)$ represents the ordinary differential equations [37]; and $f\left({\hat{u}}_{\theta }\left(X;\theta \right),X_{n},t\right)$ represents the algebraic equations [37]. The ODEs and algebraic equations were utilized as an additional regularization term [38]. By minimizing the loss function of the physics-based equations, we could incorporate the laws of physics into the NN [38,39,40]. 

When used for computing partial differential equations (PDE), the $L_{F}\left(\theta \right)$ in PINN is typically used to penalize the degree to which the model violates physical laws. However, the PDE-based $L_{F}\left(\theta \right)$ was not applicable in the continuous time modeling and prediction problems addressed in this paper. The physical laws of vehicle dynamics models are often subject to ODEs and algebraic equations. To address this, we drew inspiration from the approach of a physics-constrained neural network (PCNN) [39,40] and neural ordinary differential equations (NODEs) [41,42]. PCNN is a specific variant of PINN that introduces a regularization parameter to control the trade-off between data and knowledge-based regularization [43]. In NODEs, the hidden layers of a neural network are treated as the states of an ODE, and an ODE solver is used to compute the evolution of these states. Specifically, we incorporated ODEs to represent the dynamic evolution of the system and used algebraic equations to represent the vehicle dynamics model.

The PINN module provided the signals of the pseudo-states, which represented the rate of change of the state variable $\dot{x}$ in the LTI state space. The inputs and outputs of the PINN module were denoted as:

(12) $$X^{\left(1-22\right)}=\left[a_{x},a_{y},a_{z},\omega_{x},\omega_{y},\omega_{z},v_{x},v_{y},v_{x},\varphi ,\Theta ,\psi ,M_{y_{l}},M_{y_{r}},F_{x_{fl}},F_{x_{rl}},F_{y_{fl}},F_{y_{rl}},d_{E},d_{N},d_{U},\delta_{s}\right]\;{{\hat{u}}_{\theta }\left(X;\theta \right)}^{\left(1-6\right)}=[a_{x},a_{y},a_{z},\omega_{x},\omega_{y},\omega_{z}]$$

where $d_{E},\;d_{N},\;\mathrm{and}\;d_{U}$ are the navigation coordinates minus the current vehicle coordinates [44]. 

The ordinary differential equations were represented as:

(13) $$g_{q}\left({\hat{u}}_{\theta }\left(X;\theta \right),X_{n},z_{t}\right)=X_{n}^{\left(q+6\right)}+{{\hat{u}}_{\theta }\left(X;\theta \right)}^{\left(q\right)}\times d_{t}-z_{t}^{\left(q\right)},q=1,\;2,\;\ldots ,\;6\;g_{7}\left({\hat{u}}_{\theta }\left(X;\theta \right),X_{n},z_{t}\right)=\sum_{i=1}^{3}{[X}_{n}^{\left(i+6\right)}\times d_{t}+\frac{{{{\hat{u}}_{\theta }\left(X;\theta \right)}^{\left(i\right)}\times d_{t}}^{2}}{2}]-z_{t}^{\left(7\right)}$$

where $X_{n}^{\left(7-12\right)}=[v_{x_{n}},v_{y_{n}},v_{z_{n}},\varphi_{n},\Theta_{n},\psi_{n}]$ are the initial states of ODE outputs; $z_{t}^{\left(1-6\right)}$= $\left[v_{x_{t}},v_{y_{t}},v_{z_{t}},\varphi_{t},\Theta_{t},\psi_{t}\right]$ are the measurements of ODE outputs at timestamp t; and $d_{t}$ is the time interval between n and t. By minimizing $g_{1}\sim g_{6}$, the pseudo-states could incorporate information from the related vehicle states. The state $z_{t}^{\left(7\right)}$ represents the position change of the vehicle, which was represented as:

(14) $$z_{t}^{\left(8,9,10\right)}=[d_{E_{t}},d_{N_{t}},d_{U_{t}}]\;z_{t}^{\left(7\right)}=\sqrt{{\left(E^{t}-E^{n}\right)}^{2}+{\left(N^{t}-N^{n}\right)}^{2}+{\left(U^{t}-U^{n}\right)}^{2}}=\sqrt{{\left(d_{E_{t}}\right)}^{2}+{\left(d_{N_{t}}\right)}^{2}+{\left(d_{U_{t}}\right)}^{2}}$$

where [E,N,U] are the outputs of variable $y_{t}$ in the LTI state-space vehicle model. By minimizing $g_{7}$, the physical knowledge of $y_{t}$ was incorporated into the physics-informed loss function. The position-updated formula of the vehicle dynamics [45] may affect the learning effectiveness of the neural network. Therefore, we used the Euler integral to calculate the displacement of the vehicle. By utilizing the loss calculation method of PINN, the ODE vehicle dynamics could be combined with a data-driven model to consider multiple sensor sources.

Algebraic equations capture simple dependence relationships among vehicle states [37]. The algebraic equations representing the linear dynamics in the LTI state space could be written as $\dot{x}=AX_{n}+B_{n}$. By minimizing the loss of ${\hat{u}}_{\theta }\left(X;\theta \right)-\dot{x}$, we could incorporate the physical dynamics model into the physics-informed loss function. The referenced vehicle dynamics model was based on the two-degree-of-freedom (2-DOF) vehicle dynamics model [23,45]. To simplify the vehicle dynamics [23], we decoupled the longitudinal and latitudinal dynamics by neglecting the influence of the latitudinal and longitudinal forces.

The algebraic equation of $f_{8}\left({\hat{u}}_{\theta }\left(X;\theta \right),X_{n}\right)$ was represented as:

(15) $$f_{8}\left({\hat{u}}_{\theta }\left(X;\theta \right),X_{n}\right)=a_{x_{n}}^{\left(1\right)}-{{\hat{u}}_{\theta }\left(X;\theta \right)}^{\left(1\right)}\;X_{n}^{\left(7,13,14\right)}=[v_{x_{n}},M_{y_{l,n}},M_{y_{r,n}}]\;ma_{x_{n}}^{\left(1\right)}=F_{e_{n}}+F_{b_{n}}+F_{s_{n}}+F_{f_{n}}-F_{D_{n}}\;a_{x_{n}}^{\left(1\right)}=\frac{1}{m}(k_{1}\times M_{y_{l,n}}+k_{2}\times M_{y_{r,n}}+mg\gamma +mg\mu -k_{D}v_{x_{n}}^{2}$$

where $a_{x_{n}}^{\left(1\right)}$ is calculated based on the vehicle longitudinal dynamics; $F_{e_{n}}$ represents the transmitted force of the vehicle; $F_{b_{n}}$ represents the brake force of the vehicle; m is the gross vehicle mass; and $M_{y_{l,n}}$ and $M_{y_{r,n}}$ represent the y-axis torque of the two front wheels. We assumed $F_{e_{n}}$ and $F_{b_{n}}$ were transmitted to the front wheels and converted into $M_{y_{l,n}}$ and $M_{y_{r,n}}$, respectively (the experimental car was a front-wheel drive vehicle). Therefore, the transmitted and brake forces were calculated using the least squares method. Additionally, a small slope angle was assumed. Therefore, the dissipative force was $F_{s_{n}}=0$ and the frictional force was $F_{f_{n}}$= mgμ. Here, g represented the gravitational constant and μ was the road–wheel static friction coefficient. The air drag force $F_{D_{n}}$ was calculated based on the longitudinal velocity and the drag coefficient $k_{D}$. The parameters $k_{1}$, $k_{2}$, μ, and $k_{D}$ were calculated using the least squares method to realize online learning of the parameters.

In this paper, the tire force was directly measured. This allowed for the direct calculation of the longitudinal acceleration $a_{x_{n}}^{\left(2\right)}$ from the measured tire force. The algebraic equation of $a_{x_{n}}^{\left(2\right)}$ was represented as:

(16) $$f_{9}\left({\hat{u}}_{\theta }\left(X;\theta \right),X_{n}\right)=a_{x_{n}}^{\left(2\right)}-{{\hat{u}}_{\theta }\left(X;\theta \right)}^{\left(1\right)}\;X_{n}^{\left(15,16\right)}=[F_{x_{fl,n}},F_{x_{fr,n}}]\;a_{x_{n}}^{\left(2\right)}=\frac{{ℷ}_{1}}{m}\left(F_{x_{fl,n}}+F_{x_{fr,n}}\right)$$

where $F_{x_{fl,n}}\;\mathrm{and}\;F_{x_{fr,n}}$ are the front left and right wheel longitudinal forces, respectively; $a_{x_{n}}^{\left(2\right)}$ represents the longitudinal acceleration, ${ℷ}_{1}$ is the parameter (${ℷ}_{1}=\frac{F_{x_{fl,1}}+F_{x_{fr,1}}}{{ma}_{x_{1}}}$), and [$F_{x_{fl,1}},F_{x_{fr,1}},a_{x_{1}}$] represents the known measurement data.

The algebraic equation of $f_{10}\left({\hat{u}}_{\theta }\left(X;\theta \right),X_{n}\right)$ was calculated from the vehicle latitudinal dynamics, which was represented as:

(17) $$f_{10}\left({\hat{u}}_{\theta }\left(X;\theta \right),X_{n}\right)=a_{y_{n}}^{\left(1\right)}-{{\hat{u}}_{\theta }\left(X;\theta \right)}^{\left(2\right)}\;X_{n}^{\left(7,8,12,22\right)}=[v_{x_{n}},v_{y_{n}},\omega_{z_{n}},\delta_{s_{n}}]\;\delta_{f_{n}}=\frac{1}{i}\delta_{s_{n}}\;ma_{y_{n}}^{\left(1\right)}=F_{y_{fl,n}}+F_{y_{fr,n}}+F_{y_{rl,n}}+F_{y_{rr,n}}\;a_{y_{n}}^{\left(1\right)}=\frac{1}{m}[\frac{-2Cv_{y_{n}}-2C\left(l_{f}-l_{r}\right)\omega_{z_{n}}}{v_{x_{n}}}-mv_{x_{n}}\omega_{z_{n}}+2C\delta_{f_{n}}]$$

where C represents the tire cornering stiffness; $l_{f}\;\mathrm{and}\;l_{r}$ represent the distances from the center of the vehicle’s mass to the front and rear axles, respectively; $\omega_{z}$ denotes the yaw rate; $F_{y_{fl,n}},F_{y_{fr,n}},F_{y_{rl,n}},\;\mathrm{and}\;F_{y_{rr,n}}$ denote the lateral tire forces at the front left/right and rear left/right wheels, respectively; $\delta_{s_{n}}$ represents the steering wheel angle; $\delta_{f_{n}}$ represents the front wheel steering angle; and i denotes the function of the variable steering gear ratio [46]. This was a linear tire model and we assumed the vehicle had equal cornering stiffness for all four wheels.

Similar to $a_{x_{n}}^{\left(2\right)}$, we directly calculated the latitudinal acceleration $a_{y_{n}}^{\left(2\right)}$ from the measured tire force. The algebraic equation of $a_{y_{n}}^{\left(2\right)}$ was represented as:

(18) $$f_{11}\left({\hat{u}}_{\theta }\left(X;\theta \right),X_{n}\right)=a_{y_{n}}^{\left(2\right)}-{{\hat{u}}_{\theta }\left(X;\theta \right)}^{\left(2\right)}\;X_{n}^{\left(17,18\right)}=[F_{y_{fl,n}},F_{y_{rl,n}}]\;a_{y_{n}}^{\left(2\right)}=\frac{{ℷ}_{2}}{m}(F_{y_{fl,n}}+F_{y_{fr,n}})$$

where $F_{y_{fl,n}}\;\mathrm{and}\;F_{y_{fr,n}}$ represent the front left and right wheel latitudinal forces, respectively; $a_{y_{n}}^{\left(2\right)}$ represents the latitudinal acceleration; and ${ℷ}_{2}$ is the parameter (${ℷ}_{2}=\frac{F_{y_{fl,1}}+F_{y_{fr,1}}}{{ma}_{y_{1}}}$).

The algebraic equation of $f_{12}\left({\hat{u}}_{\theta }\left(X;\theta \right),X_{n}\right)$ was calculated from the kinematic vehicle model, which was represented as:

(19) $$f_{12}\left({\hat{u}}_{\theta }\left(X;\theta \right),X_{n}\right)=\omega_{z_{n}}^{\left(1\right)}-{{\hat{u}}_{\theta }\left(X;\theta \right)}^{\left(6\right)}\;\beta_{n}=\tan^{-1}[\frac{l_{r}}{l_{f}+l_{r}}\tan(\delta_{f_{n}})]\;\omega_{z_{n}}^{\left(1\right)}=\frac{v_{x_{n}}}{l_{f}}\times \sin(\beta_{n})$$

where $\beta_{n}$ represents the vehicle slip angle. 

The physics-based equations presented in this paper, ($g_{1-7}\left({\hat{u}}_{\theta }\left(X;\theta \right),X_{n},z_{t}\right)$ and $f_{8-12}\left({\hat{u}}_{\theta }\left(X;\theta \right),X_{n}\right)$), were applicable within the LTI state space. This implied that the pseudo-states were assumed to remain constant over the time interval [n+1, n+2, …, n+F]. This assumption ensured the validity and applicability of the equations mentioned earlier, allowing for the incorporation of physics-based constraints into the neural network model.

The PINN module utilized the PyTorch deep learning framework. To consider the dynamics of different states, the Adam optimizer with a 0.001 learning rate was used to train the network. The model was trained using a Nvidia GTX 3080Ti GPU.

### 3.3. The UKF-M Module

The UKF-M is a novel UKF on manifolds, with versatility that allows direct application to numerous practical manifolds. For stochastic processes on Riemannian manifolds, the theory of Lie groups [47] is used to define the vehicle's attitude estimation. The IMU-GNSS sensor-fusion model [34] was a UKF-M-based filter, which is a standard 3D kinematic model based on inertial inputs. The UKF-M algorithm utilized in PINN UKFM was based on the methodology proposed in [34]. The modification of PINN UKFM involved using the outputs of the PINN module to replace the original IMU measurements.

The states of a moving vehicle in a discrete dynamic system are represented as:

(20) $$\chi_{n}\in M=\{C_{n}\in R^{3\times 3},v_{n}\in R^{3},P_{n}\in R^{3},b_{g_{n}}\in R^{3},b_{a_{n}}\in R^{3}\}$$

where $\chi_{n}$ denotes the state of a vehicle belonging to a parallelizable manifold M; n is the current timestamp; $v_{n}=(v_{E_{n}},v_{N_{n}},v_{U_{n}})$ is the velocity vector ($v_{E_{n}}$–velocity east and $v_{N_{n}}$-velocity north); $P_{n}=(E_{n},\;N_{n},H_{n})$ is vehicle coordinate in the navigation coordinates; $b_{g_{n}}=(b_{\omega_{x,\;n}},b_{\omega_{y,\;n}},b_{\omega_{z,\;n}})$ is the gyro bias; $b_{a_{n}}=(b_{a_{x,\;n}},b_{a_{y,\;n}},b_{a_{z,\;n}})$ is the accelerometer bias; and $C_{n}$ is a special orthogonal group that represents 3D rotation [47].

(21) $$SO\left(3\right):=\{C_{n}\in R^{3\times 3}|C_{n}{C_{n}}^{T}=1,\det C_{n}=1\}$$

where 1 is the identity matrix. Based on the time derivative of $C_{n}{C_{n}}^{T}=1$, a skew-symmetric matrix $C_{n}^{T}{\dot{C}}_{n}$ was obtained:

(22) $${C_{n}}^{T}{\dot{C}}_{n}+{{\dot{C}}_{n}}^{T}C_{n}=0$$

The $C_{n}^{T}{\dot{C}}_{n}$ as a skew-symmetric matrix is often noted as ${\left[\omega \right]}_{\times }\;$:

(23) $${C_{n}}^{T}{\dot{C}}_{n}={\left[\omega \right]}_{\times }=\left[\begin{array}{ccc} 0 & -\omega_{z} & \omega_{y}\\ \omega_{z} & 0 & -\omega_{x}\\ -\omega_{y} & \omega_{x} & 0 \end{array}\right]$$

where ${\left[\omega \right]}_{\times }$ is in the Lie algebra of $SO\left(3\right)$. The Lie algebra is a vector space and can be decomposed into:

(24) $${\left[\omega \right]}_{\times }=\omega_{x}\left[\begin{array}{ccc} 0 & 0 & 0\\ 0 & 0 & -1\\ 0 & 1 & 0 \end{array}\right]+\omega_{y}\left[\begin{array}{ccc} 0 & 0 & 1\\ 0 & 0 & 0\\ -1 & 0 & 0 \end{array}\right]+\omega_{z}\left[\begin{array}{ccc} 0 & -1 & 0\\ 1 & 0 & 0\\ 0 & 0 & 0 \end{array}\right]$$

where $\omega =[\omega_{x},\omega_{y},\omega_{z}]$ is in the vector of angular velocities. For the ω constant, we obtained the ODE solution:

(25) $$C_{n}=\exp\left({\left[\omega \right]}_{\times }n\right)$$

where exp() is the exponential map on the SO(3) [42] and $C_{0}=I.$ The exp() map was derived from the time derivatives of $\chi_{n}\in M$. The vector fields were defined as:

(26) V1Cn=Cn100^,V2Cn=Cn010^,V3Cn=Cn001^

where ^ is the hat map [47], and $V_{1}$, $V_{2},$ and $V_{3}$ are the vector fields. 

Similar to the Gaussian belief of the Kalman filter, the UKF-M algorithm builds a probability distribution as $\chi_{n}\sim N({\hat{\chi }}_{n},P_{n})$ for the random variable $\chi_{n}\in M$ as:

(27) $$\chi_{n}=\varphi \left({\hat{\chi }}_{n},\xi_{n}\right),\xi_{n}\sim N(0,P_{n})$$

where ${\hat{\chi }}_{n}$ is viewed as the mean estimate at timestep n; φ is the propagation function; $\xi_{n}\in R^{d}$ is a random Gaussian vector; N is the Gaussian distribution; and $P_{n}\in R^{d\times d}$ is the covariance matrix. $\varphi \left({\hat{\chi }}_{n},\xi_{n}\right)$ ∈M is obtained by starting from ${\hat{\chi }}_{n}$ and integrating the vector field $\sum_{i=1}^{d}\xi_{n}^{i}V_{i}$ (*d* is the dimension of the associated vector fields).

Consider that the probability distribution of $\chi_{n}$ is $p(\chi_{n})$. The additional information about $\chi_{n}$ is obtained from the measurement $y_{n}$ as:

(28) $$y_{n}=h\left(\chi_{n}\right)+v_{n}$$

where h is the observation function, and $v_{n}\sim$ $N(0,R_{n})$ denotes the white Gaussian noise. The UKF-M module used the gyro measurements and acceleration as inputs to update the random variable χ. The measurements of this standard 3D kinematic model were represented as:

(29) $$y_{n}=\{\mu_{n}\in R^{3},a_{b_{n}}\in R^{3}\}$$

where $\mu_{n}=(\omega_{x_{n}},\omega_{y_{n}},\omega_{z_{n}})$ represents the gyroscope, and $a_{b_{n}}=(a_{x_{n}},a_{y_{n}},a_{z_{n}})$ denotes the accelerometer. The UKF-M algorithm [34] updated the state and covariance by combining measurements $y_{n}$ and system states $\chi_{n}$.

In PINN UKFM, the output of the PINN module was utilized to filter the noise and minimize the norm errors. Pseudo-states ${\hat{u}}_{\theta }$ served as the calibrated IMU measurements for the filtering process. The states of the pseudo-states were represented as:

(30) $$y_{n}={y_{n}}^{\prime }={\hat{u}}_{\theta }=\{\mu_{n}\in R^{3},a_{b_{n}}\in R^{3}\}$$

where ${y_{n}}^{\prime }={\hat{u}}_{\theta }$ represents the PINN module output as the pseudo-states.

Using the propagation function [34], PINN UKFM built the vehicle model. First, the gyro measurements were inputted to calculate the rotation matrix:

(31) $$C_{n+1}=C_{n}\exp\left(\left(\mu_{n}-b_{g_{n}}+w_{n}^{\left(0:3\right)}\right)\times d_{t}\right)$$

where exp is the exponential map on the SO(3), and $d_{t}$ is the integration step. In this vehicle model, $w_{n}^{\left(0:12\right)}$ represented the noise, and $w_{n}^{\left(0:3\right)}$, $w^{\left(3:6\right)}$, $w^{\left(6:9\right)},$ and $w^{\left(9:12\right)}$ were the submatrices of $w_{n}^{\left(0:12\right)}$. Next, the vehicle acceleration was inputted to calculate the calibrated vehicle acceleration:

(32) $$a=C_{n}\left(a_{b_{n}}-b_{a_{n}}+w^{\left(3:6\right)}\right)+g$$

where g=[0,0,−9.82] represents the gravitational constant and a represents the calibrated vehicle acceleration. Based on the calibrated vehicle acceleration, the model updated the vehicle speed as:

(33) $$v_{n+1}=v_{n}+ad_{t}$$

where $v_{n}=(v_{E_{n}},v_{N_{n}},v_{U_{n}})$ denotes the velocity vector. Based on the vehicle velocity vector, the model updated the vehicle position vector as:

(34) $$P_{n+1}=P_{n}+v_{n}\times d_{t}+\frac{a\times {d_{t}}^{2}}{2}$$

where $P_{n}=(E_{n},\;N_{n},U_{n})$ is the vehicle coordinates in the navigation coordinates. Finally, the model uploaded the gyro and accelerometer biases as:

(35) $$b_{a,n+1}=b_{a,n}+w^{\left(6:9\right)}\times d_{t}\;b_{g,n+1}=b_{g,n}+w^{\left(9:12\right)}\times d_{t}$$

where $b_{g,n}$ represents the gyro bias, and $b_{a,n}$ represents the accelerometer bias.

The probability distribution of χ and the propagation function remained unchanged; therefore, the posterior distribution $p\left(\chi_{n}|{y_{n}}^{\prime }\right)$ was calculated as $p\left(\chi_{n}|y_{n}\right)$. The pseudo-states ${y_{n}}^{\prime }$ provided information about the sigma point $\xi_{n}$. First, the sigma points $\xi_{n}$ were computed as:

(36) $$\xi_{j_{𝓷}}=\left\{\begin{array}{c} {\mathrm{col}\left(\sqrt{\left(\lambda +d\right)P_{n}}\right)}_{j},j=1,\ldots ,d\\ -{\mathrm{col}\left(\sqrt{\left(\lambda +d\right)P_{n}}\right)}_{j},j=d+1,\ldots ,2d \end{array}\right.\;\lambda =(\alpha^{2}-1)d$$

where λ is the scale parameter [48]; α is a free parameter chosen by the practitioner [49] (α must be small); *d* is the dimension of the associated vector fields; $P_{n}$ is the covariance matrix at timestep n; and col represents that the j column of the matrix is the weight associated with the j point. Second, these sigma points passed through the vehicle model to yield the set of transformed sigma point $y_{j_{n}}$:

(37) $$y_{j_{n}}=\left\{\begin{array}{c} h\left(\varphi \left({\hat{\chi }}_{n},0\right)\right),j=0\\ h\left(\varphi \left({\hat{\chi }}_{n},\xi_{j_{n}}\right)\right),j=1,\ldots ,2d \end{array}\right.$$

where $h\left(\varphi \left({\hat{\chi }}_{n},0\right)\right)$ is the unnoisy state model, h is the observation function (as in Equations (31–35)), and ${\hat{\chi }}_{n}$ is the prior mean estimate of the current state. Third, the mean and covariance of the transformed sigma points [30] were computed as:

(38) $$\bar{y_{n}}=\varpi_{m}y_{0_{n}}+\sum_{j=1}^{2d}\varpi_{j}y_{j_{n}}\;P_{y_{n}y_{n}}=\sum_{j=0}^{2d}\varpi_{j}(y_{j_{n}}-\bar{y_{n}}){\left(y_{j_{n}}-\bar{y_{n}}\right)}^{T}+R_{n}\;P_{\xi_{n}y_{n}}=\sum_{j=1}^{2d}\varpi_{j}\xi_{j_{n}}(y_{j_{n}}-\bar{y_{n}})\;\varpi_{m}=\frac{\lambda }{\lambda +d}\;,\;\varpi_{j}=\frac{1/2}{\lambda +d}\;$$

where $\bar{y_{n}}$ represents the mean of the transformed sigma points; $P_{y_{n}y_{n}}$ is the covariance of the transformed sigma points; $P_{\xi_{n}y_{n}}$ is the cross-covariance of the transformed sigma points; $\varpi_{m}$ and $\varpi_{j}$ are weights; and $R_{n}$ is the covariance matrix of white Gaussian noise. Next, PINN UKFM employed the Kalman updated equation to update the state and covariance as:

(39) $$K_{n}=P_{\xi_{n}y_{n}}P_{y_{n}y_{n}}^{-1}\;{{\hat{\chi }}_{n}}^{+}=\varphi \left({\hat{\chi }}_{n},K_{n}({y_{n}}^{\prime }-\bar{y_{n}})\right)\;{P_{n}}^{+}=P_{n}-K_{n}P_{y_{n}y_{n}}{K_{n}}^{T}$$

where ${y_{n}}^{\prime }$ is the PINN module output (pseudo-states), $K_{n}$ is the gain matrix, ${{\hat{\chi }}_{n}}^{+}$ is the posterior mean estimate state, $P_{n}$ is the prior estimate covariance matrix of the current state, and ${P_{n}}^{+}$ is the posterior estimate covariance matrix. 

The unscented transformation [50] was employed to approximate the posterior $p\left(\xi_{n}|{y_{n}}^{\prime }\right)$ for $\xi_{n}$ as:

(40) $$p\left(\xi_{n}|{y_{n}}^{\prime }\right)\sim N\left(\bar{\xi_{n}},{P_{n}}^{+}\right)\;\bar{\xi_{n}}=K_{n}({y_{n}}^{\prime }-\bar{y_{n}})$$

where $\bar{\xi_{n}}$ represents the noise-free mean. 

The unscented approximation to the posterior $p\left(\xi_{n}|{y_{n}}^{\prime }\right)$ was thus the distribution of a Gaussian $\bar{\xi_{n}}+{\xi_{n}}^{+}$ with ${\xi_{n}}^{+}\sim N\left(0,{P_{n}}^{+}\right)$ [34]. Then, PINN UKFM approximated the posterior distribution $p\left(\chi_{n}|{y_{n}}^{\prime }\right)$ as:

(41) $$\chi_{n}\approx \varphi \left({{\hat{\chi }}_{n}}^{+},{\xi_{n}}^{+}\right),\;{\xi_{n}}^{+}\sim N\left(0,{P_{n}}^{+}\right)\;{{\hat{\chi }}_{n}}^{+}=\varphi \left({\hat{\chi }}_{n},\bar{\xi_{n}}\right)\;\chi_{n}\approx \varphi \left(\varphi \left({\hat{\chi }}_{n},\bar{\xi_{n}}\right),{\xi_{n}}^{+}\right)$$

where ${\xi_{n}}^{+}$ represents the posterior noise. The posterior distribution $p\left(\chi_{n}|{y_{n}}^{\prime }\right)$ boiled down to a Bayesian estimation problem [47] that incorporated the information from the PINN module. 

In this paper, we focused on describing how PINN UKFM updated the state estimation ${{\hat{\chi }}_{n}}^{+}$ and covariance matrix ${P_{n}}^{+}$ when pseudo-states ${y_{n}}^{\prime }$ arrived. Additionally, the UKF-M algorithm could propagate the state without sensor measurement [34], which was implemented in our program. 

## 4. Experimental Results

### 4.1. Vehicle Platform

**Figure 4:**
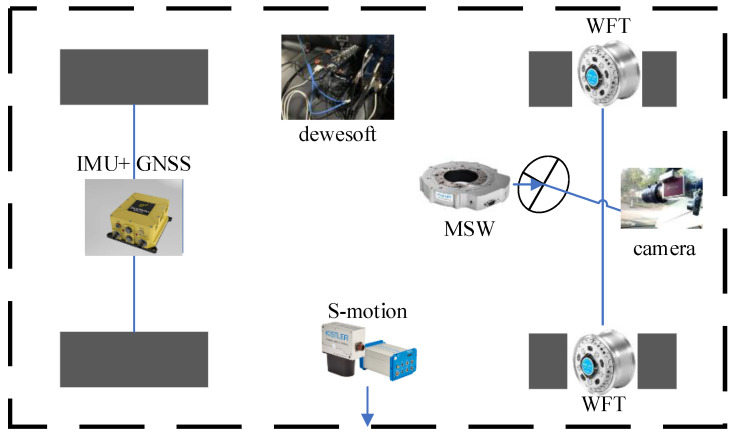
Hardware configuration.

A vehicle platform was built to validate the proposed algorithm. The hardware configuration is shown in Figure 4. The sensors included GNSS, IMU, MSW, WFT, S-Motion, and a camera. D[WE-43A-USB and Dewesoft SIRIUS acquisition systems were used. The DEWE-43A-USB system obtained the S-Motion and MSW signals through high-speed CAN channels, while the Dewesoft SIRIUS system collected the WFT signals through high-speed CAN channels. A computer was used for all data acquisition.

**Figure 5:**
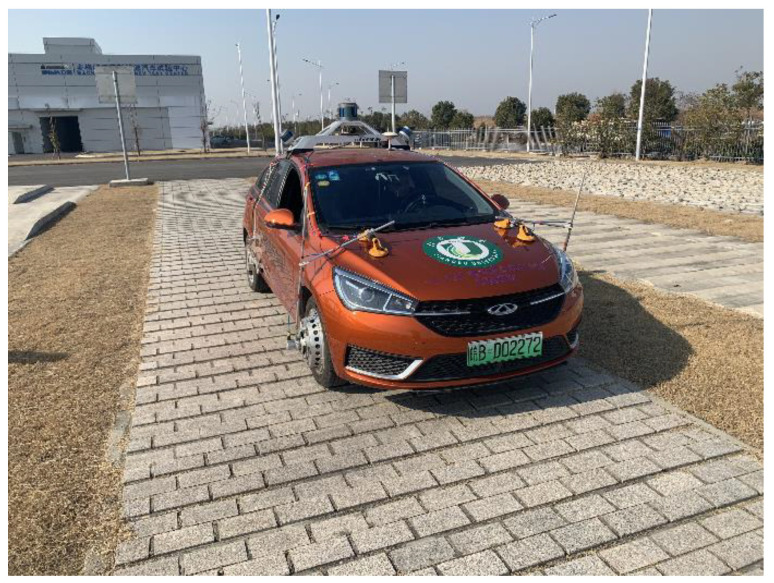
The test vehicle.

First, the Mako G-192B monocular camera [51], produced by the Allied Vision Company, captured image data of the environment. Then, the starNeto XW-GI [52] provided the IMU and GNSS data, using differential methods to measure both position and attitude. $GPFPD was selected as the starNeto communication protocol to acquire signals for [$E,N,U,\varphi ,\Theta ,\psi ,v_{e},v_{n},v_{z}$]. In the third step, the S-Motion system, produced by Kistler, provided signals for $\left[v_{x},a_{x},v_{y},a_{y},a_{z},\omega_{x},\omega_{y},\omega_{z}\right]$. In the fourth step, the MSW by Kistler was used to measure the steering wheel angle, speed, and torque. Finally, the WFT by Kistler was used to measure the wheel forces and moments under dynamic conditions for the left and right front wheels. The WFT provided signals for $\left[F_{x},M_{x},F_{y},M_{y},F_{z},M_{z}\right]$ in the front wheels and three axes of WFT, similar to the vehicle body coordinates. The hardware implementation in the test vehicle [52,53,54,55,56] is shown in Figure 5. 

**Figure 6:**
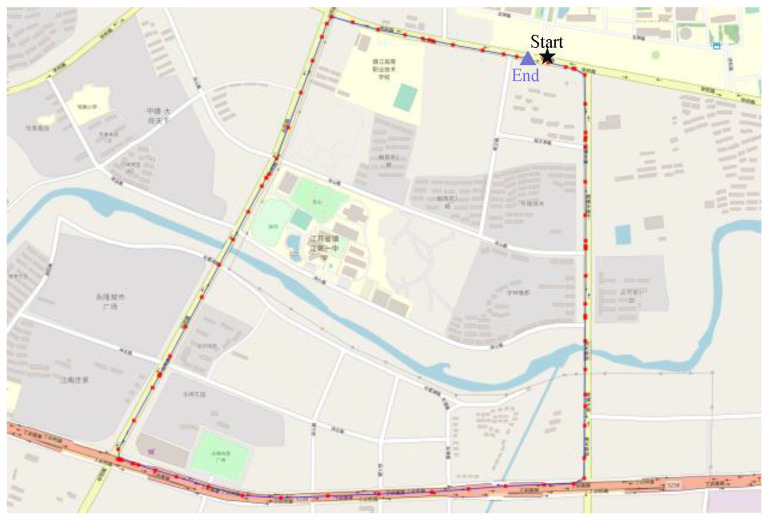
The trajectory of the experiment.

The test was conducted on cement pavement at Jiangsu University, and the vehicle trajectory is shown in Figure 6. The weather during the test was rainy, which reduced the tire–road friction coefficient and increased the nonlinearity of the vehicle's dynamic relationships. A total of 85% of the data were used for training and to verify the PINN module, while the remaining 15% were reserved for testing. The dataset included scenarios such as an overpass (high speed), a school (low speed), a slope, traffic lights, and vehicle turning. The road scene could be changed by altering the dataset splits. In the experiment, the last 15% of the dataset was used to evaluate the performance of PINN UKFM.

### 4.2. Parameter Settings and Training of All Comparative Methods

PINN: The structure of the PINN was chosen as {22, 32, 32×5, 64, 128, 128, 64, 32, 6}. For easy calculation of $v_{z}$, we subtracted the gravity vector from the acceleration $a_{z}$ during data processing [34]. The layer {22, 32} was the encoder layer, and the layers {32, 32×5, 64, 128, 128, 64, 32} were the temporal interaction layers. The layer {32, 6} was the decoder layer. The prediction states of the PINN module were the IMU calibration values. 

subPINN: To address the influence of loss backward during training, a single-output scheme of the PINN was implemented. The vehicle dynamics/kinematic models were used to build the data-driven model $\left[a_{x},a_{y},\omega_{z}\right]$. This subPINN had the same structure as the PINN and a single output. Specifically, for training $\left[a_{z},\omega_{x},\omega_{y}\right],$ the structure {1, 2, 32, 32, 64} was used to reduce the computational complexity and improve accuracy. The inputs of $\left[a_{z},\omega_{x},\omega_{y}\right]$ were [$a_{z}$,$v_{z}$], [$\omega_{x}$,φ], and [$\omega_{y}$,Θ], respectively. Different from the UTM coordinates, the attitudes did not differ greatly, so they were directly inputted here.

MLP [24,25,57]: The multilayer perceptron (MLP) is a commonly used approach for building data-driven vehicle dynamics models. The structure of the MLP used in this study was inspired by [24] and was chosen as {22, 32, 32×5, 64, 128, 128, 64, 32, 6}. The ReLU activation function was applied between each layer. Different from the approach used in [24], we used the layers {32, 32×5} as the temporal interaction layers, which is a common approach in data-driven dynamics modeling [25].

LSTM [24,57,58]: Long Short-Term Memory (LSTM) is another classic method used to build data-driven dynamics models, which is employed for predicting the acceleration and angular velocity of vehicles [57,58]. LSTM is known for its ability to capture temporal dependencies in the data. In this study, LSTM was implemented with a general transformation structure as {22, 32, 128, 32, 6}, where {22, 32} and {32, 6} represented the MLP layers. The exponential linear unit (ELU) [35] function was applied after the first layer, and {32, 32, 128} represented the two LSTM layers.

Physical models [23,45]: The yaw rate formulas based on longitudinal/lateral dynamics and kinematic models were used in this paper. The longitudinal dynamics parameters were calculated using the least squares method [23]. The lateral dynamics parameters were computed based on the actual vehicle parameters ($l_{f},l_{r},m$) and the relationships between states ($C=\frac{F_{y}}{a_{y}}$). We assumed the yaw rate was an LTI state. Therefore, a kinematic model [45] was used to calculate the yaw rate.

The wheel force [$F_{x},F_{y}$] measured by WFT could be utilized to calculate the acceleration through algebraic equations. Additionally, we used the linearly constrained least squares method to combine these dynamics as:

(42) $$ma_{x_{n}}=\left(K_{1}\times M_{y_{l,n}}+K_{2}\times M_{y_{r,n}}+K_{3}mg-K_{4}v_{x_{n}}^{2}\right)+K_{5}F_{x_{fl,n}}+K_{6}F_{x_{fr,n}}+K_{7}\;ma_{y_{n}}=K_{8}\left(\frac{-4Cv_{y_{n}}-2C\left(l_{f}-l_{r}\right)\omega_{z_{n}}}{v_{x_{n}}m}-mv_{x_{n}}\omega_{z_{n}}+\frac{2C\delta_{n}}{m}\right)+K_{9}F_{y_{fl,n}}+K_{10}F_{y_{fr,n}}+K_{11}$$

where $K_{1}{\sim K}_{6},K_{8}{\sim K}_{10}$ represent the parameters of the least squares method, and $K_{7}\;\mathrm{and}\;K_{11}$ represent the coefficients of the least squares method. 

**Figure 7:**
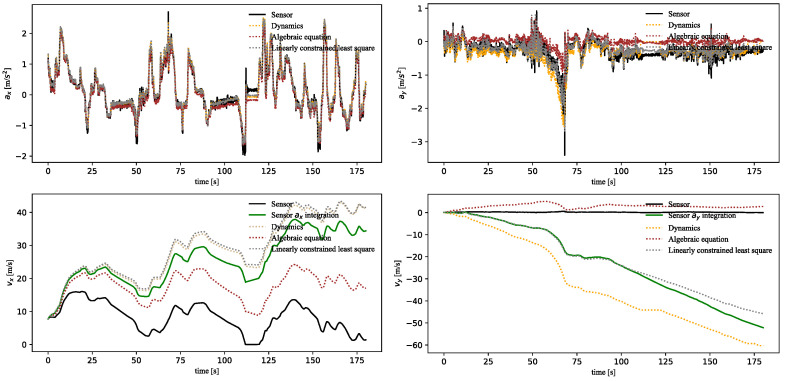
The results of physical models.

The resulting states of the physical models are illustrated in Figure 7. In the experiment, we not only compared the prediction states with the sensor signals but also compared the integration states $\left[v_{x},v_{y},v_{z},\varphi ,\Theta ,\psi \right]$ with the integrated sensor signals, which could be expressed as:

(43) $$Z_{t}=Z_{0}+\int_{i=0}^{t}u_{i}dt$$

where $Z_{0}$ is initial state, t is the timestamp, and $u_{i}=[a_{x},a_{y},a_{z},\omega_{x},\omega_{y},\omega_{z}]$ are the states at timestamp i.

As shown in Figure 7, the “Algebraic equation” represented the calculation results of Equations (16) and (18). The “Linearly constrained least square” represented the calculation results of Equation (42). By combining the forces and dynamics, the output of this equation provided a better fit to the IMU measurements. This method could fit multiple sensors to improve the accuracy of the dynamics, when the IMU measurements were accurate. However, due to the IMU drift, the integration error of this model was relatively large. The “Dynamics” represented the longitudinal/lateral dynamics model based on Equations (15) and (17), which is commonly used for vehicle control. In this paper, “Linearly constrained least square” and “Dynamics” were established based on the IMU measurement data. Therefore, the results of these models were close to the IMU measurements but also had large integration errors. 

To maintain visual clarity, we only used the vehicle dynamics (“Dynamics”) and kinematic models for comparison. MLP and LSTM also utilized the PyTorch deep learning framework. We employed the Adam optimizer with a 0.001 learning rate to train the network. The models were trained using the Nvidia GTX 3080Ti GPU. All models, including PINN, subPINN, MLP, and LSTM, were trained using the same training dataset. However, there was a difference in the loss calculation method. Different from the loss calculation method established by PINN, MLP and LSTM used the IMU measurements to build data-driven vehicle models [25,57,58]. These methods commonly assumed that the ground truth states of the vehicle were accessible. In this paper, we used highly accurate S-motion data as a surrogate for the actual vehicle state for MLP and LSTM training. 

### 4.3. Validation of the PINN Module

In this subsection, we compare the PINN module with the MLP, LSTM, and vehicle dynamics/kinematic models and vehicle measurements. These data-driven models were trained using the dataset described in Section 4.1. The root mean square error (RMSE) [24,59] was used to evaluate the performance of the models, which was represented as:

(44) $$\mathrm{RMSE}=\sqrt{\frac{1}{S}\sum_{i=1}^{S}{\left(\varkappa_{i}-\hat{\varkappa_{i}}\right)}^{2}}$$

where S = 1800 is the predicted steps, $\varkappa_{i}=[u_{i},Z_{i}]$ represents the i-th value of the predicted results, and $\hat{\varkappa_{i}}$ represents the i-th value of the reference results (sensor measurements). 

Table 2 presents the RMSEs of the predicted states using different methods. The results demonstrated that except for longitudinal/lateral acceleration, the PINN-based approaches (PINN and subPINN) estimated the vehicle state more accurately. The inferior prediction performance on longitudinal/lateral acceleration was attributed to sensor drift. By integrating the acceleration to obtain velocity, it was shown that the PINN-based methods could effectively take the influence of other sensors and realize IMU calibration.

**Figure 8:**
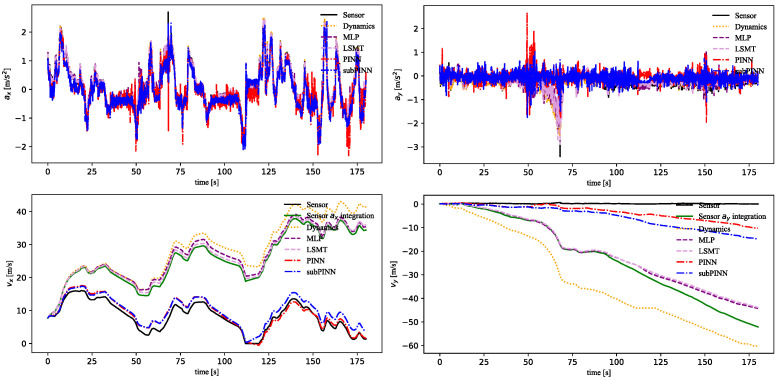
The prediction results of states ${[a}_{x},{v_{x},a_{y},v}_{y}]$ and sensor measurements.

As shown in Figure 8, the estimation results of ${[a}_{x},{v_{x},a_{y},v}_{y}]$ were obtained. “Sensor $a_{x}$ integration” was the ODE states inferred from the original signals using Equation (43) and is shown in green. The PINN model incorporated both longitudinal/lateral vehicle dynamics and LTI-based vehicle displacement, which yielded higher-precision results than the subPINN model. Compared with the MLP and LSTM models, the PINN model had better prediction accuracy of the integrated states. This was due to the PINN model being able to incorporate more dynamics knowledge into the data-driven model.

**Figure 9:**
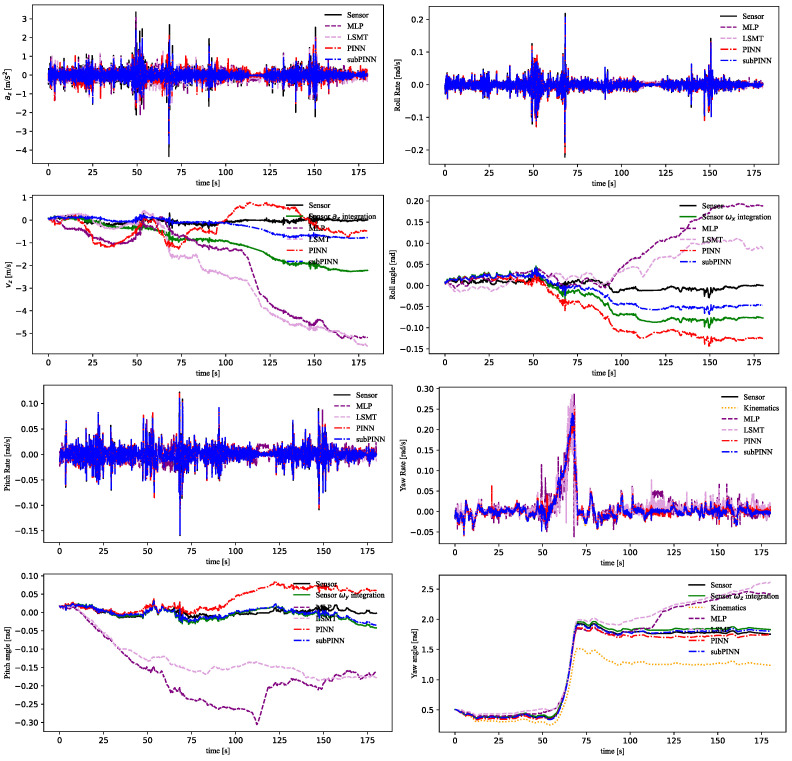
The prediction results of states ${[a}_{z},v_{z},\omega_{x},\varphi ,\omega_{y},\Theta ,\omega_{z},\psi ]$ and sensor measurements.

The estimation results of states ${[a}_{z},v_{z},\omega_{x},\varphi ,\omega_{y},\Theta ,\omega_{z},\psi ]$ were obtained as shown in Figure 9. The experiment of ${[a}_{z},\omega_{x},\omega_{y}$] aimed to reduce the noise in acceleration and angular velocity by combining the PINN module with the linear ODEs. As the noise impact on the S-Motion sensor increased over time, the subPINN model demonstrated superior accuracy in estimating the state variables while maintaining the sensor data characteristics and reducing noise. Both the PINN and subPINN models used the vehicle kinematic model in modeling $\omega_{x}$. The subPINN model converged more readily in yaw rate prediction than the other models. Compared to the MLP and LSTM models, the subPINN model had better prediction accuracy in each state.

The subPINN model estimated a single vehicle state, which simplified the training process and enabled easier convergence during training. In contrast, the training process of the PINN model was more complex and it was difficult to achieve optimal results. The subPINN model offered advantages in terms of training efficiency and high accuracy results. This gave the subPINN model a certain attractiveness for engineering implementations. However, the subPINN model could not establish connections between multiple states like the PINN model in order to achieve more comprehensive modeling. Therefore, the PINN model performed better in terms of longitudinal/lateral dynamics accuracy. 

### 4.4. Validation of PINN UFM 

The experimental results presented above demonstrated the advantages of the PINN module, but some issues in the comparison still remained. The vehicle states were not connected through a vehicle-based model during the comparison, which failed to reflect the impact of the estimation states on the vehicle dynamics. Compared to other vehicle states, the cm-level GNSS positioning could better reflect the effectiveness of the presented estimation states.

**Figure 10:**
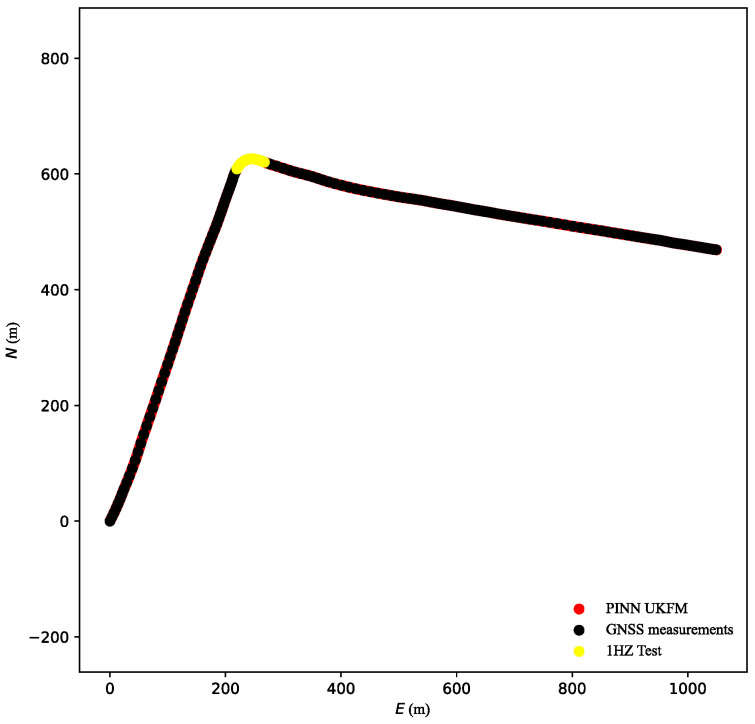
Compared trajectories at 20 Hz GNSS position.

In this subsection, the UKF-M module based on the output of the PINN module is demonstrated. This module reduced state noise through the Gaussian noise hypothesis. Figure 10 shows the trajectories of PINN UKFM and sensor inputs. The vectors [E, N, h] were entered at 20 Hz. The covariance matrices of the point were iterated quickly, which could not encapsulate the performance improvement of PINN UKFM.

**Figure 11:**
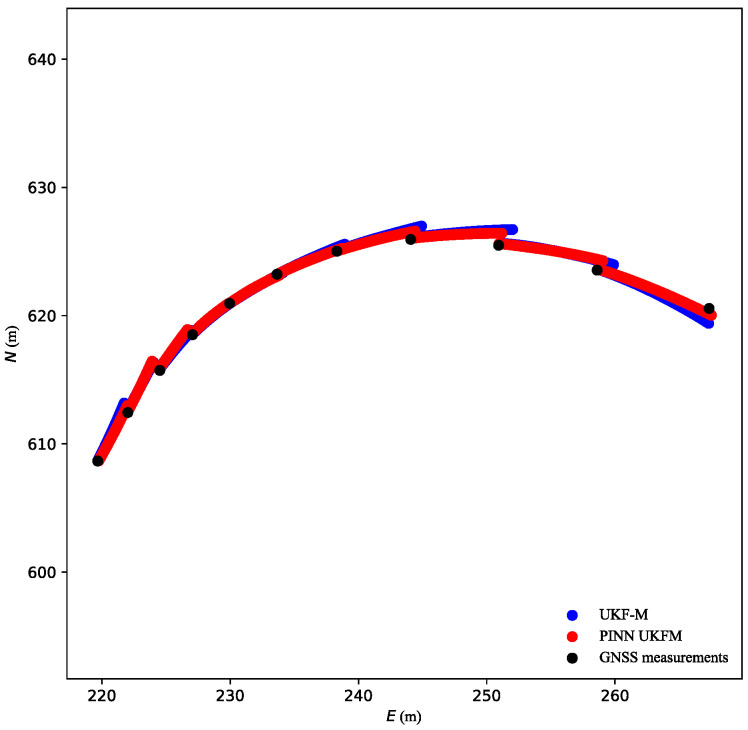
Compared trajectories at 1 Hz GNSS position.

Therefore, the frequency of the trajectory coordinates was reduced to 1 Hz through downsampling. As illustrated in Figure 11, the results obtained using PINN UKFM were superior to those obtained using the conventional UKF-M algorithm. Without coordinate input, the vehicle coordinates were updated in the vehicle model using the UKF-M module. The PINN module could reduce the influence of vehicle sensor drift and provided a reliable state estimation result that was closer to the true state of the vehicle. Therefore, the updated vehicle position of PINN UKFM was also closer to the true vehicle state.

**Figure 12:**
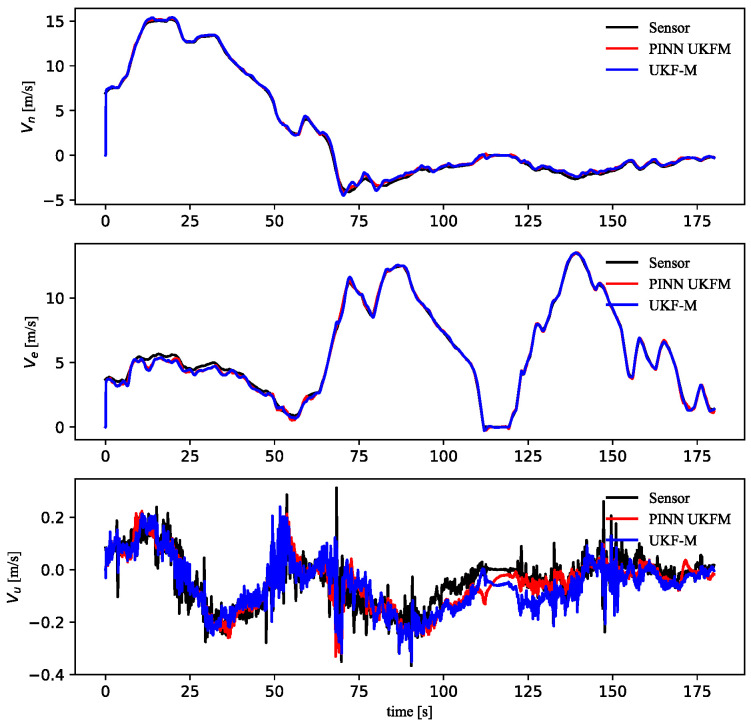
Compared 3D velocities at 20HZ GNSS position.

Additionally, the UKF-M module could estimate the vehicle speed, which included $\left[v_{e},v_{n},v_{u}\right]$. However, the $\left[v_{e},v_{n},v_{u}\right]$ were not inputted into the PINN module. Next, the modeling results were compared to the measurement states to verify PINN UKFM correctness. As shown in Figure 12, compared to the UKF-M algorithm, PINN UKFM could better estimate $v_{u}$.

**Figure 13:**
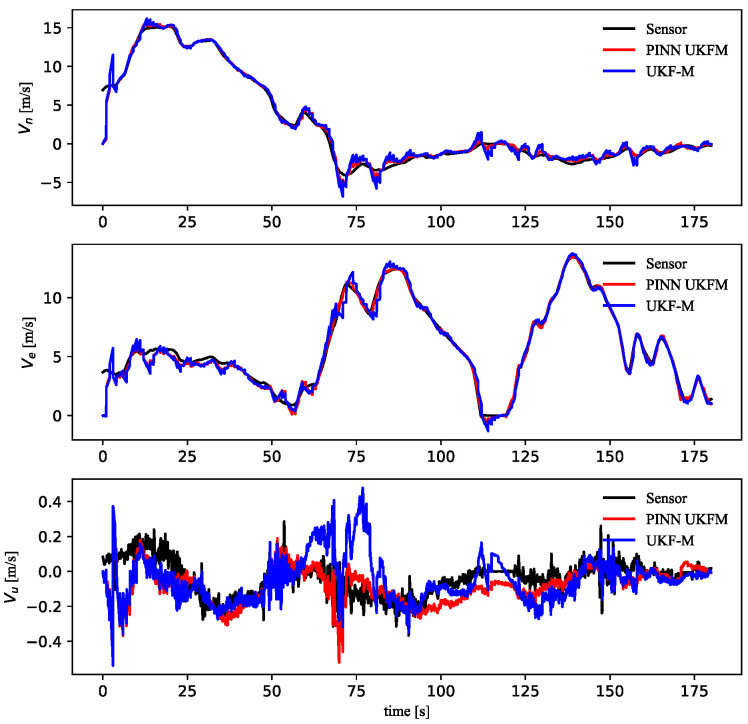
Compared 3D velocities at 1HZ GNSS position.

Considering that the cm-level GNSS positioning has a great impact on the covariance matrix, the velocity at the 1 Hz GNSS position was also compared, as shown in Figure 13. The PINN UKFM results were significantly better than the UKF-M results. 

Evidently, PINN UKFM exhibited remarkable performance in vehicle state estimation, although there was still potential for further improvement. A possible enhancement lies in leveraging the signals from the CAN bus to replace the WFT and MSW sensors. The CAN bus grants access to a wealth of vehicle sensor data, including valuable information such as the steering angle, throttle opening, and brake pressure. By incorporating these signals as inputs to the model, a dynamic vehicle model could be constructed, enabling more accurate state estimation. Additionally, the S-Motion sensor can also be replaced by a cheaper IMU. Using more universal CAN bus signals and IMUs, the model could be implemented on more vehicle platforms without being limited by specific sensors.

## 5. Conclusions

In this study, a novel method for vehicle state estimation combining PINN and UKF-M has been proposed. The sensor module collects various sensor signals and feeds them into the PINN module. The PINN module automatically calibrates the IMU by utilizing the ODE relationships that exist between multiple sensors. The PINN-based model mitigates sensor errors and facilitates sensor fusion, leveraging the LTI hypothesis for loss calculation in order to reduce noise and bias in the original sensor data. It exhibits clear advantages in multi-sensor fusion compared to existing data-driven vehicle dynamics models. The resulting pseudo-states are then used as inputs to the UKF-M module to model the vehicle trajectory. The experimental results confirmed its effectiveness, with the PINN module successfully eliminating IMU drift and the PINN UKFM trajectory exhibiting less deviation from the cm-level GNSS positioning than the UKF-M trajectory. In the future, we aim to explore more cost-effective solutions to implement the PINN UKFM method. Additionally, the authors intend to use PINN UKFM in OCCS control.

## Figures and Tables

**Table 1:** The sensor inputs of PINN UKFM.

Sensor Types	Signal Name	Symbol	Units
GNSS	Easting	E	m
GNSS	Northing	N	m
GNSS	Altitude	U	m
GNSS	UTM velocity	$V_{gps}$	m/s
IMU	Roll angle	φ	rad
IMU	Pitch angle	Θ	rad
IMU	Yaw angle	ψ	rad
IMU	Vertical velocity	$v_{z}$	m/s
S-Motion	Longitudinal velocity	$v_{x}$	m/s
S-Motion	Longitudinal acceleration	$a_{x}$	$m/s^{2}$
S-Motion	Lateral velocity	$v_{y}$	m/s
S-Motion	Longitudinal acceleration	$a_{y}$	$m/s^{2}$
S-Motion	Vertical acceleration	$a_{z}$	$m/s^{2}$
S-Motion	Roll rate	$\omega_{x}$	rad/s
S-Motion	Pitch rate	$\omega_{y}$	rad/s
S-Motion	Yaw rate	$\omega_{z}$	rad/s
WFT	Wheel force	$F_{x},F_{y},F_{z}$	kN
WFT	Wheel torque	$M_{x},M_{y},M_{z}$	kN·m
MSW	Steering wheel angle	$\delta_{s}$	rad

**Table 2:** The RMSEs of the predicted states using different methods.

Model	$a_{x}$	$a_{y}$	$a_{z}$	$\omega_{x}$	$\omega_{y}$	$\omega_{z}$
MLP	0.1117	0.1524	0.2729	0.0164	0.0171	0.0174
LSTM	0.1167	0.1444	0.2626	0.0171	0.0175	0.0167
PINN	0.2733	0.3904	0.2806	0.0110	0.0084	0.0058
subPINN	0.2301	0.3606	0.2167	0.0076	0.0049	0.0036
Model	$v_{x}$	$v_{y}$	$v_{z}$	φ	Θ	ψ
MLP	20.2365	25.2757	2.7479	0.1027	0.1865	0.3334
LSTM	19.6955	24.8413	0.5609	0.0613	0.1391	0.3856
PINN	1.3007	4.6707	0.5609	0.0806	0.0429	0.0475
subPINN	1.8171	7.3559	0.4169	0.0312	0.0113	0.0217

## Data Availability

The dataset from our experiments and model can be downloaded in Google drive. Moreover, we have developed a Jupyter notebook (script/PINN_UKFM_TEST.ipynb) that enables online access to all experimental results. https://drive.google.com/drive/folders/1iKkIuy6L8LUOVyypgxCLsdL_lAH1mCjG, accessed on 1 June 2023.
